# Vegetative and Fecundity Fitness Benefit Found in a Glyphosate-Resistant *Eleusine indica* Population Caused by 5-Enolpyruvylshikimate-3-Phosphate Synthase Overexpression

**DOI:** 10.3389/fpls.2021.776990

**Published:** 2021-11-19

**Authors:** Zhiling Li, Xiangju Li, Hailan Cui, Guodong Zhao, Dan Zhai, Jingchao Chen

**Affiliations:** ^1^Institute of Plant Protection, Chinese Academy of Agricultural Sciences, Beijing, China; ^2^School of Life Sciences, Inner Mongolia University, Hohhot, China; ^3^College of Plant Protection, Henan Agricultural University, Zhengzhou, China

**Keywords:** fitness, benefit, glyphosate, *EPSPS*, metabonomics, auxin

## Abstract

Fitness is an important trait in weed species that have developed herbicide resistance, including resistance to the popular herbicide glyphosate. Fitness cost is commonly found in weeds with glyphosate resistance, which is caused by target-site mutations. In this study, the vegetative and fecundity fitness traits in a glyphosate-resistant (GR) *Eleusine indica* population caused by 5-enolpyruvylshikimate-3-phosphate synthase (*EPSPS*) overexpression were investigated under glyphosate-free conditions. The results showed that the resistance index of the population resistant (R) to glyphosate compared with that of the population susceptible (WT) to it was approximately 4.0. Furthermore, *EPSPS* expression level in the R plants was 20.1–82.7 times higher than that in the WT plants. The dry weight of the R population was significantly higher than that of the WT population at the later growth stage after planting; a similar trend was observed for leaf area. In addition, seed production in the R population was 1.4 times higher than that in the WT population. The R and WT populations showed similar maximum germination rates and T_50_ values. UPLC-MS/MS was performed for the metabolic extracts prepared from the leaves of R and WT populations to address changes in the metabolome. A total of 121 differential metabolites were identified between R and WT individuals. The levels of 6-hydroxy-1H-indole-3-acetamide and indole acetaldehyde, which are associated with auxin synthesis, were significantly higher in plants of the R population than in those of the WT population. However, some secondary metabolite levels were slightly lower in the R population than in the WT population. To conclude, in this study, vegetative and fecundity fitness benefits were found in the GR *E. indica* population. The results of metabolome analysis indicate that the increase in 6-hydroxy-1H-indole-3-acetamide and indole acetaldehyde levels may be the result of fitness benefit. Further studies should be conducted to confirm the functions of these metabolites.

## Introduction

Glyphosate is a non-selective herbicide that has been extensively used worldwide for weed control in crop and non-crop situations (Duke and Powles, 2008). This herbicide clearly inhibits the target 5-enolpyruvylshikimate-3-phosphate synthase (*EPSPS*; E.C. 2.5.1.19), which plays an important role in the formation of aromatic amino acids (Steinrücken and Amrhein, 1980). The annual Gramineae weed goosegrass [*Eleusine indica* (L.) Gaertn.], originated from Asia, is now widely distributed in temperate and tropical regions (Holm et al., 1977). This weed can lead to a 20–50% reduction in the yield of infested crops (Ma et al., 2015; Song et al., 2019). Glyphosate is the most commonly used herbicide to control weed species, including goosegrass, in infested acreage, especially orchards (Duke and Powles, 2008). However, after many years of selection pressure, weed species have evolved to develop resistance to glyphosate; 54 glyphosate-resistant (GR) weed species have been reported (Heap, 2021). In China, most of the GR goosegrass species have been reported in orchards; in recent years, GR goosegrass has been reported to occur in cotton fields and tea gardens, as well as in direct sowing rice fields (Chen et al., 2015a, 2020a,b; Deng et al., 2020).

One or more amino acid substitution in the conserved regions of *EPSPS* or overexpression of this target gene is considered the cause of target-site resistance (TSR) to glyphosate in weeds (Sammons and Gaines, 2014). The mutation commonly occurs at positions Thr102 or Pro106, and *EPSPS* overexpression is commonly caused by the amplification of this gene in the chromosome (Gaines et al., 2010; Yu et al., 2015; Li et al., 2018; Perotti et al., 2019). Non-target-site resistance (NTSR) is another resistance mechanism that is associated with increased metabolism, reduced translocation or uptake, and enhanced vacuolar sequestration of glyphosate (Délye, 2013). For goosegrass, both target site mutations at amino acid residues and *EPSPS* overexpression have been reported (Baerson et al., 2002; Chen et al., 2015b; Yu et al., 2015). In addition, our previous study has found that *EPSPS* overexpression and Pro-106-Ala mutation have evolved in the same GR individuals (Chen et al., 2020b).

Fitness is defined as the reproductive success of a plant in a given environment; therefore, seed production, germination rate, and vegetative growth are important parameters that determine fitness (Vila-Aiub et al., 2009). Fitness cost is a characteristic of herbicide-resistant weeds, where plants show a competitive disadvantage in the absence of herbicide selection pressure (Vila-Aiub et al., 2009). The GR weed species, caused by either NTSR or TSR mechanisms, are associated with fitness costs (Yanniccari et al., 2015; Fernández-Moreno et al., 2017; Vila-Aiub et al., 2019). A GR *Lolium perenne* population, due to high *EPSPS* activity, showed a 33% reduction in leaf blade area and a 55% reduction in shoot biomass, compared with susceptible plants (Yanniccari et al., 2015). A *Lolium multiflorum* population exhibited altered uptake and translocation, which reduced seed output by at least 38% compared with that of the susceptible population (Fernández-Moreno et al., 2017). Except for the fitness cost, high fecundity rate (30% increase in silique and seed number per plant) was found in GR *Oryza sativa* and *Arabidopsis thaliana*, in which transgenic technology was used to overexpress the endogenous gene *EPSPS* (Yang et al., 2017; Fang et al., 2018). A mutated *EPSPS* gene which was overexpression in *O. sativa* produced 17%–19% more grains compared to the wild type in the absence of glyphosate application (Achary et al., 2020). For goosegrass, a significant fitness cost occurred in the GR population with a double mutation in *EPSPS* (Han et al., 2017). However, there have been few reports on fitness traits in GR goosegrass with *EPSPS* overexpression. Here, the characteristics of fitness of a GR goosegrass population caused by *EPSPS* overexpression, as confirmed in our previous study, was assessed. The objectives of this research were to: (1) confirm the resistance level and mechanism for the selected population, (2) to investigate vegetative growth, seed production, and germination rate of resistant versus susceptible plants sharing a common genetic background, and (3) to elucidate the differences in metabolites between resistant and susceptible plants using metabolomics.

## Materials and Methods

### Plant Material and Genetic Background Control

We selected a GR goosegrass population caused by *EPSPS* overexpression, as confirmed in our previous study (Chen et al., 2015b). To minimize variability in genetic backgrounds, we selected resistant and susceptible individuals from within a population (Vila-Aiub et al., 2015; Keshtkar et al., 2019). In detail, seeds from more than 100 individuals in the resistant population were collected and then grown to the tillering stage under (27 ± 4)/(20 ± 4)°C day/night temperature with a 14 h photoperiod (Chen et al., 2020b). At least 40 seedlings were cloned into two groups and labeled. One group was sprayed with glyphosate at a dosage of 900 g ai ha^–1^ to detect resistant and susceptible plants. Twenty-one days after treatment, based on the response of the corresponding clones, the individuals of the other group not treated with glyphosate were classified. The individuals for which sensitivity was confirmed were grown for seed production in two isolated places to prevent cross-pollination. The seeds were collected for fitness studies and named R (resistant) and WT (susceptible).

### Excluding the Non-target Site Resistance Mechanism

Seeds of the R and WT populations were cultured in plastic pots (8 cm × 8 cm) and kept in a greenhouse (Beijing, China) under the same condition described above. All individuals were thinned to eight plants per pot around the two-leaf stage and watered as needed (Chen et al., 2020b). To confirm the *EPSPS* overexpression resistance mechanism and no other NTSR mechanisms exist in R population, different treatments were conducted for these two populations. One group treated a set of doses (0, 56, 112, 225, 450, and 900 g ai ha^–1^ for WT population; 0, 450, 900, 1800, 3600, and 7200 g ai ha^–1^ for R population) of glyphosate (Roundup, isopropylamine salt of glyphosate, 410 g ae L^–1^, Bayer Crop Science, St. Louis, MO, United States). All the individuals were applied glyphosate at four- to six-leaf stage using a moving TeeJet^®^ XR8002 flat fan nozzle cabinet sprayer (Beijing Research Center for Information Technology in Agriculture, Beijing, China) (Wang et al., 2018). For the other group, the P450 inhibitor piperonylbutoxide (10 mM) and the GST inhibitor 4-chloro-7-nitro-1,2,3-benzoxadiazole (3 mM) were sprayed for all individuals before glyphosate treatment for 3 and 72 h, respectively. Twenty-one days after treatment, fresh weight of the shoots in each pot under different treatments was measured (Jugulam and Shyam, 2019; Chen et al., 2020b).

### Expression of 5-Enolpyruvylshikimate-3-Phosphate Synthase

The expression level of *EPSPS* was measured in these two populations using a standard method. In brief, leaf tissue of 24 individuals from each population was collected when the plants were at the five- to six-leaf stage. Total RNA was extracted from each sample, and the quality was detected using agarose gel electrophoresis. First-strand complementary DNA was synthesized using *EasyScript*^®^ All-in-One First-Strand cDNA Synthesis SuperMix (TransGen Biotech, Beijing, China). We used confirmed primer pairs to amplify *EPSPS*, with chloroplast acetolactate synthase (*ALS*) as the reference gene (Chen et al., 2017a). Quantitative real-time PCR (qPCR) was conducted in a 20 μL reaction with the 2× SYBR Green PCR master mix and run on an ABI 7500 PCR machine (Applied Biosystems Inc., Foster City, CA, United States). The expression of the target gene relative to that of the reference gene was analyzed using the 2^–ΔΔCt^ method and the formula −2^ΔCt^ = −[(Ct, *EPSPS* − Ct, *ALS*)_treat_ − (Ct, *EPSPS* − Ct, *ALS*)_control_] (Livak and Schmittgen, 2001). This experiment was repeated twice.

### Assessment of Vegetative and Fecundity Traits Without Competition

The purified seeds of R and WT were cultured using the method described above, and only one individual was maintained in each pot. At 20, 45, and 70 days after the individuals emerged, leaf area for all leaves was measured using a leaf area scanner (YMJ-B, Zhejiang Top Instruments Co., Ltd., China). After measuring the leaf area, all above ground tissues were dried at 80°C; the dry shoot biomass was measured after 48 h (Ghanizadeh and Harrington, 2019). The seed number of 24 individuals was also detected from each population at the maturity stage. This experiment was repeated twice.

### Characterization of Seedling Emergence Rate

Thirty seeds each of the R and WT populations, were placed on a 9 cm × 9 cm petri dish lined with filter paper and moistened with 5 mL distilled water. They were kept in a growth chamber with a 16 h photoperiod; 21 μmol m^–2^ s^–1^ light intensity; and day/night temperatures of 30 and 20°C, respectively. Seedling emergence (appearance of the coleoptile) was recorded daily for 10 days and this experiment was repeated twice (Matzrafi et al., 2017).

### Metabolites Analysis Using LC-MS/MS

Five individuals each of the R and WT populations were selected, and the leaf tissue was prepared using a standard method. In brief, 50 mg of sample was ground using liquid nitrogen and homogenized using an extraction solution with an isotopically labeled internal standard mixture. The samples were then incubated for 1 h at −40°C and centrifuged at 12,000 rpm at 4°C for 15 min. The resulting supernatant was transferred to a fresh glass vial for further analysis.

LC-MS/MS analyses were performed using a UHPLC system (Thermo Fisher Scientific, Waltham, MA, United States) with a UPLC HSS T3 column (2.1 mm × 100 mm, 1.8 μm) coupled to a Q Exactive HFX mass spectrometer (Orbitrap MS, Thermo Fisher Scientific, CA, United States). The mobile phase consisted of 5 mmol L^–1^ ammonium acetate and 5 mmol L^–1^ acetic acid in water (A) and acetonitrile (B). The auto-sampler temperature was 4°C, and the injection volume was 3 μL. A standard method for QE HFX mass spectrometry was performed, and all parameters were the same as those used in the previous study (Li et al., 2021).

### Statistical Analyses

The data from the whole-plant assay and seedling emergence analysis were analyzed using the log-logistic model: *Y* = *y*_0_ + *a*/[1 + (*X*/*X*_0_)*^b^*], using SigmaPlot 12.0 (Systat Software, San Jose, CA, United States) (Seefeldt and Fuerst, 1995; Ritz et al., 2013). In this model, *b* is the slope of the curve, *y*_0_ is the lower limit, *a* is the difference between the upper and lower limits, and *X*_0_ is the herbicide dose required for 50% plant growth reduction (GR_50_) or the time required for 50% of the seeds to be germinated (T_50_). The resistance index (RI) reflects the resistance level for the R population and is the ratio of the GR_50_ of the R population to that of the WT population.

5-Enolpyruvylshikimate-3-phosphate synthase expression and fitness trait data were analyzed using Student’s *t*-test for R and WT populations. All analyses were performed using SPSS (version 13.0; SPSS, Chicago, IL, United States). The raw data from metabolite analysis were converted to the mzXML format using ProteoWizard software and processed with an in-house program, which was developed using R package and based on XCMS, for peak detection, extraction, alignment, and integration. Then, an in-house MS2 database (BiotreeDB) was used for metabolite annotation. The cutoff for annotation was set at 0.3. Principal component analysis (PCA) and orthogonal projections to latent structures discriminant analysis (OPLS-DA) were performed using the SIMCA software (V16.0.2, Sartorius Stedim Data Analytics AB, Umea, Sweden). Metabolites with variable importance in the projection (VIP) values >1 and a false discovery rate <0.05 were considered differentiated (Zhang et al., 2020).

## Results

### Response of R and WT Populations to Glyphosate

**FIGURE 1:**
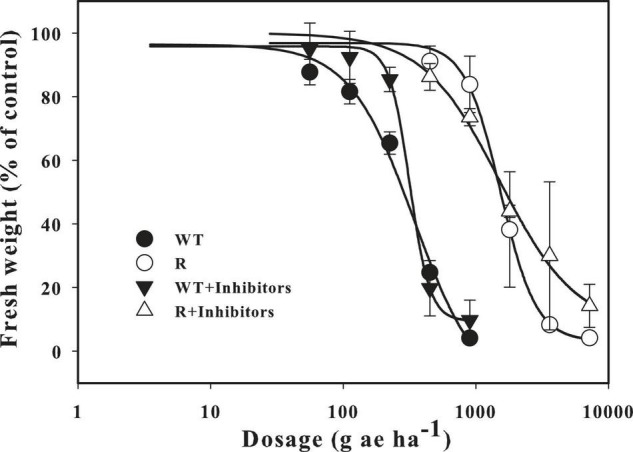
The response of resistant goosegrass populations (R) and the WT population to different doses of glyphosate under the condition of treated with P450s and GSTs inhibitors or not. Vertical bars represent standard errors of the mean (SEM).

The P450 inhibitor piperonylbutoxide and the GST inhibitor 4-chloro-7-nitro-1,2,3-benzoxadiazole were used to identify possible NSTR in the R population. The response to glyphosate was similar between resistant individuals that were treated with the inhibitors and those that were not. A slight growth inhibition was observed after administration of the recommended dose (Figure 1). The GR_50_ value for glyphosate calculated from the fresh weights of the R population was 1539.4 g ae ha^–1^, and a similar result (1517.0 g ae ha^–1^) was calculated after treatment with the inhibitors. Compared with the WT population, the RI of R to glyphosate was 4.0 and 4.8, respectively.

### Expression Level of 5-Enolpyruvylshikimate-3-Phosphate Synthase

**FIGURE 2:**
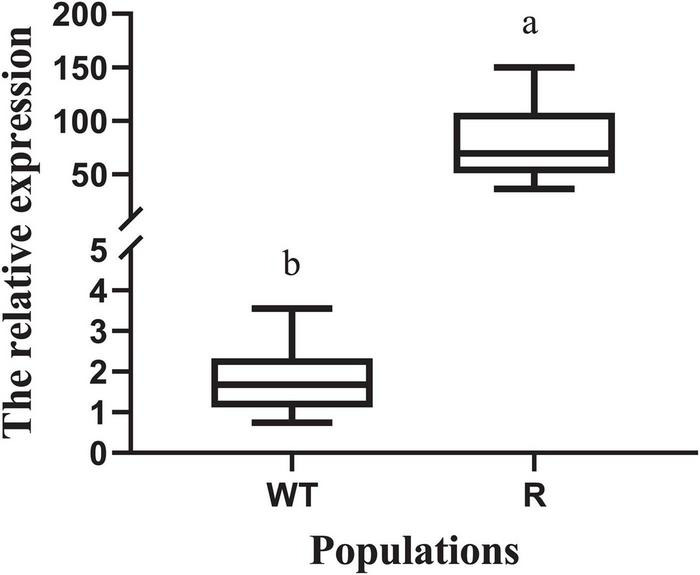
Expression levels of the target genes *EPSPS* in the plants of resistant (R) and susceptible (WT) individuals without herbicide treatment. The reference genes used for the above target genes *EPSPS* were *acetolactate synthase* (*ALS*). The different lowercase letters indicate that the parameters between the populations was significantly different by Student’s *t*-test (*P* < 0.05). Vertical bars represent standard errors of the mean (SEM).

Under glyphosate-free conditions, expression of *EPSPS* relative to that of the reference gene *ALS* was determined for the R and WT populations. The expression level of *EPSPS* (relative to that of *ALS*) ranged from 0.7 to 3.6 in the WT individuals (Figure 2). *EPSPS* expression level in R individuals showed a wide range, with the expression level in some of them reaching up to 150.0 (relative to that of *ALS*). *EPSPS* expression level in the R plants was 20.1–82.7 times higher than that in the WT plants.

### Vegetative Growth and Fecundity

**FIGURE 3:**
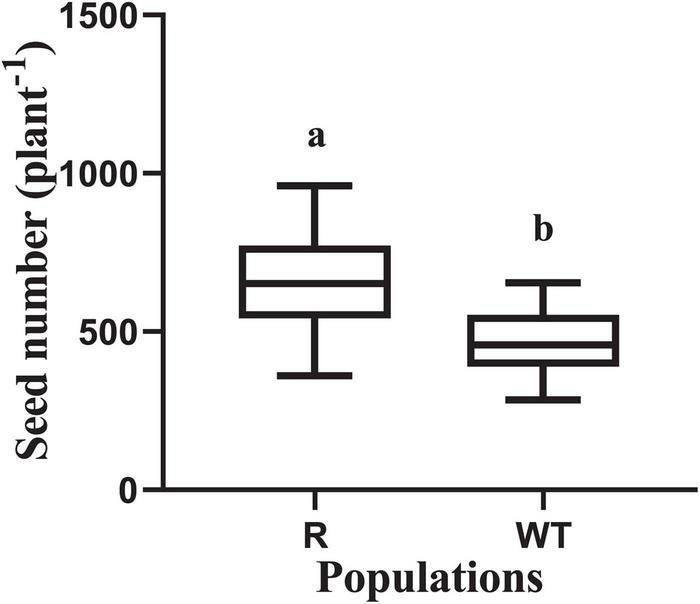
Seed number for the glyphosate-resistant goosegrass population (R) and for susceptible population (WT). Values are mean (*n* ≥ 20) and vertical bars represent the standard errors. The different lowercase letters indicate that the parameters between the populations (R and WT) was significantly different by Student’s *t*-test (*P* < 0.05).

The dry weight of above ground tissues and the total leaf area were recorded at 20, 45, and 70 days after planting. No significant differences were found in the dry weight and leaf area between the R and WT populations at 20 and 45 days after planting (Table 1). However, the dry weight of the R population was significantly higher than that of the WT population at 70 days after planting, and a similar trend was found for the leaf area. Furthermore, seed production in the R population was much higher than that in the WT population, at 658 and 466 per plant, respectively (Figure 3).

**TABLE 1:** Dry weight and leaf area of glyphosate resistant (R) and susceptible (WT) goosegrass populations harvested at 20, 45, and 70 days after planting.

Repeat	Population	Dry weight (mg plant^–1^)			Leaf area (cm plant^–1^)		
		20 days	45 days	70 days	20 days	45 days	70 days
First experiment	R	11.9a	715.0a	2041.8a	36.7a	155.2a	274.4a
	WT	9.7a	732.5a	1775.0b	37.4a	121.0a	210.9b
Second experiment	R	16.1a	620.0a	2026.4a	58.2a	148.8a	247.8a
	WT	16.6a	708.8a	1540.0b	50.0a	152.0a	186.1b

*Different letters indicate significant differences between the mean values of R and WT for each experiment according to Student’s t-test (P < 0.05).*

### Seedling Emergence Rate

**FIGURE 4:**
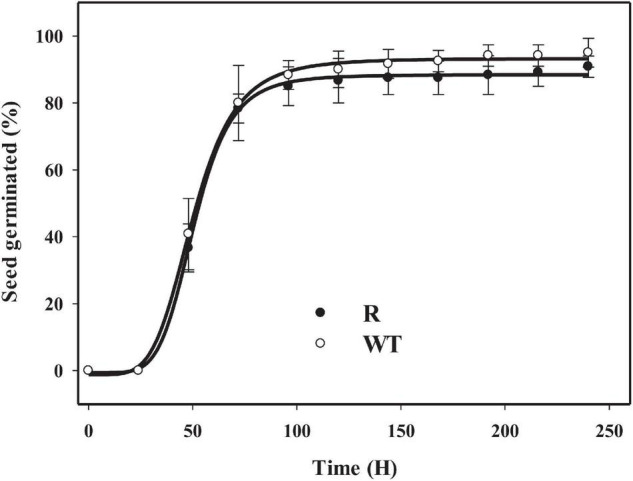
Germination results for glyphosate resistant (R) and susceptible (WT) populations in goosegrass under a condition of (30/20)°C day/night temperature with a 14 h photoperiod. Vertical bars represent standard errors of the mean (SEM).

The GR population R and susceptible population WT showed similar maximum germination rate and T_50_ under the selected conditions (Figure 4). From 24 to 72 h, the seedlings from both the R and WT populations emerged quickly and at a high percentage. At approximately 96 h, maximum germination was reached in both R and WT populations.

### Metabolites Analysis

Metabolite differences between the R and WT populations were detected using UPLC-MS/MS, and five individuals were selected from each population. The results of PCA and OPLS-DA indicate that the metabolome between R and WT shows an identical pattern (Supplementary Figure 1). The values of *R*^2^*Y* and *Q*^2^, calculated by a permutation test, were 0.97 and 0.01, respectively (Supplementary Figure 1). All the above findings indicate that the established model has no overfitting phenomenon and can be used in subsequent analyses.

A total of 13,056 metabolites were detected after preprocessing the raw data, and 3,624 of them were identified using different databases. Under the criteria of statistical significance (VIP > 1 and *p* < 0.05), 121 differential metabolites were identified between R and WT individuals (Supplementary Table 1 and Table 2). Among these metabolites, 95 were downregulated and 26 were upregulated in R relative to those in WT (Supplementary Table 1 and Table 2). The levels of metabolites associated with auxin synthesis were significantly higher in the R population than in the WT population (Table 2). However, the levels of some secondary metabolites were lower in the R individuals than in the WT individuals. In addition, metabolites in the tryptophan (Trp) and tyrosine (Tyr) metabolism pathways were significantly different between the R and WT populations (Supplementary Figure 2).

**TABLE 2:** The differentially expressed metabolites between R and WT individuals.

ID^a^	MS2 name^b^	MS2 score^c^	VIP value	*P*-value	Log_2_ fold change
221	Arbutin	0.93	2.18	0.005	3.45
382	6-Hydroxy-1H-indole-3-acetamide	0.79	1.82	0.001	2.96
224	Serotonin	0.92	2.30	0.001	2.04
305	(7alpha,10beta)-1(10->19)-abeo-7-acetoxyisoobacun-3,10-olide	0.86	1.83	0.023	1.97
160	Indole acetaldehyde	0.91	2.25	0.001	1.87
86	Mustakone	0.99	1.71	0.027	1.58
761	Coumestrin	0.46	2.24	0.000	−2.25
352	Chrysoeriol 7-O-(6″-malonyl-glucoside)	0.82	2.27	0.010	−2.13
584	Tricin arabinoside	0.63	2.25	0.002	−1.97
581	Vicenin 2	0.64	2.32	0.010	−1.85
145	Aflatoxin G	0.97	2.06	0.010	−1.79
213	Cyanidin 3-rutinoside	0.94	2.23	0.020	−1.72
639	Cyanidin 3-(3″-malonyl-glucoside)	0.59	2.14	0.009	−1.67
679	Erysodine	0.56	2.02	0.030	−1.64
55	Luteolin	1.00	2.00	0.006	−1.61
196	Trifolian	0.94	2.00	0.008	−1.59

*^a^Identity document of the metabolite in the database.*

*^b^The metabolites matched by second-stage mass spectrometry.*

*^c^The MS2 score ranged from 0 to 1 and the metabolite is matched more accurate when the MS2 score near to 1.*

## Discussion

In this study, the fitness characteristics of a GR goosegrass population (caused by *EPSPS* overexpression) were investigated in the absence of glyphosate. To estimate the fitness cost, individuals or genotypes must share the same genome, except for the gene or genes endowing resistance (Vila-Aiub et al., 2015). Creating near-isogenic lines, selecting resistant and susceptible individuals from within a population, segregating F2 populations, and conducting experiments by comparing many resistant and susceptible populations are common methods to avoid the effects of genetic background (Vila-Aiub et al., 2015; Keshtkar et al., 2019). For goosegrass, the second method is the most preferred way to eliminate the influence of genetic background and was also used in this study (Han et al., 2017; Chen et al., 2021). In addition, the mechanism of *EPSPS* overexpression was confirmed, and its expression level was 20.1–82.7 times higher in the R population than in the WT population. Similar expression levels were also found in other GR weeds that developed resistance *via* the same mechanism (Gaines et al., 2010; Malone et al., 2015; Ngo et al., 2018). Many studies use P450 and GST inhibitors to successfully explore the potential NTSR in weeds (Yuan et al., 2007; Han et al., 2021). These findings in this study suggest that no NTSR mechanisms involving P450s or GSTs exist in the R population.

At the later growth stage, a fitness benefit was found according to dry weight, leaf area, and seed production in this study. These results were similar to those of transgenic *A. thaliana* overexpressing *EPSPS via* the CaMV35S promoter, which resulted in a 30% increase in silique and seed number (Fang et al., 2018). In contrast to our results, plant growth and reproductive fitness traits in *Amaranthus palmeri* and *Kochia scoparia* were not affected by *EPSPS* overexpression, which was caused by the amplification of the gene in the genome (Vila-Aiub et al., 2014; Giacomini, 2015; Kumar and Jha, 2015). Furthermore, a fitness cost in seed weight production was found in a *K. scoparia* population with *EPSPS* amplification (Martin et al., 2017). The GR individuals of *L. perenne*, exhibiting 15-fold more *EPSPS* transcripts and 3-fold more *EPSPS* activity than the susceptible individuals, displayed a 40% reduction in the total number of seeds produced under field conditions (Yanniccari et al., 2015). Overexpression of *EPSPS* can substantially increase EPSPS enzyme production, as confirmed in our previous studies (Chen et al., 2017b). In theory, the extra copies of *EPSPS* gene, transcript, and protein may lead to material and energy expenses (Akashi and Gojobori, 2002; Lynch and Marinov, 2015). However, different fitness traits in different weed species with *EPSPS* overexpression make it more complex. Fang et al. (2018) found that a fecundity advantage occurred in a transgenic *A. thaliana* lineage over-expressing *EPSPS*. This fitness trait was associated with increased levels of the auxin indole-3-acetic acid (IAA), which plays an important role in the regulation of plant growth and development (Fang et al., 2018). Studies have indicated that Trp is the main precursor for IAA in plants, and four proposed pathways for the biosynthesis of IAA from Trp have been identified in plants (Mashiguchi et al., 2011). In this research, metabolites in the Trp metabolism pathway showed significant differences between the R and WT populations. The levels of two important precursors of IAA synthesis – namely, 6-hydroxy-1H-indole-3-acetamide and indole acetaldehyde – were significantly higher in the R population than in the WT population (Table 2). This indicates that auxin also plays an important role in imparting beneficial fitness traits to the selected R goosegrass population in this study. The high concentration of free amino acids can be regulated, and catabolism of Tyr has been shown to return the highest energy in ATP currency in plants (Hildebrandt et al., 2015). It was hypothesized that the energy cost invested in the massive *EPSPS* amplification of glyphosate-resistant weeds would be compensated by catabolism of the excess amino acids, particularly Tyr, produced by the amplified *EPSPS* activity. To support this hypothesis, in this study, Tyr in the R population was slightly lower than that in the WT population, indicating that most of the excess Tyr has been catabolized.

In this study, vegetative and fecundity fitness benefits were found in the GR *E. indica* population. The results of metabolome analysis suggest that the increase in levels of 6-hydroxy-1H-indole-3-acetamide and indole acetaldehyde, which are related to auxin synthesis, is due to glyphosate resistance. Further studies should be conducted to confirm the functions of these metabolites.

## Data Availability Statement

The original contributions presented in the study are included in the article/Supplementary Material, further inquiries can be directed to the corresponding author.

## Author Contributions

JC designed the research. ZL, GZ, and DZ performed the experimental work and the data analysis. XL and HC provided helpful suggestions for the data analysis and manuscript revision. JC and ZL wrote the manuscript. All authors read and approved the final manuscript.

## Conflict of Interest

The authors declare that the research was conducted in the absence of any commercial or financial relationships that could be construed as a potential conflict of interest.

## Publisher’s Note

All claims expressed in this article are solely those of the authors and do not necessarily represent those of their affiliated organizations, or those of the publisher, the editors and the reviewers. Any product that may be evaluated in this article, or claim that may be made by its manufacturer, is not guaranteed or endorsed by the publisher.
