# Supplementary material for: Vegetative and Fecundity Fitness Benefit Found in a Glyphosate-Resistant Eleusine indica Population Caused by 5-Enolpyruvylshikimate-3-Phosphate Synthase Overexpression
Source: Front Plant Sci. 2021 Nov 19;12:776990. doi: 10.3389/fpls.2021.776990 (PMC8639585; doi:10.3389/fpls.2021.776990)
Supplement: Supplementary file 1 [file Data_Sheet_1.zip › Supplementary Figure S1.DOCX]

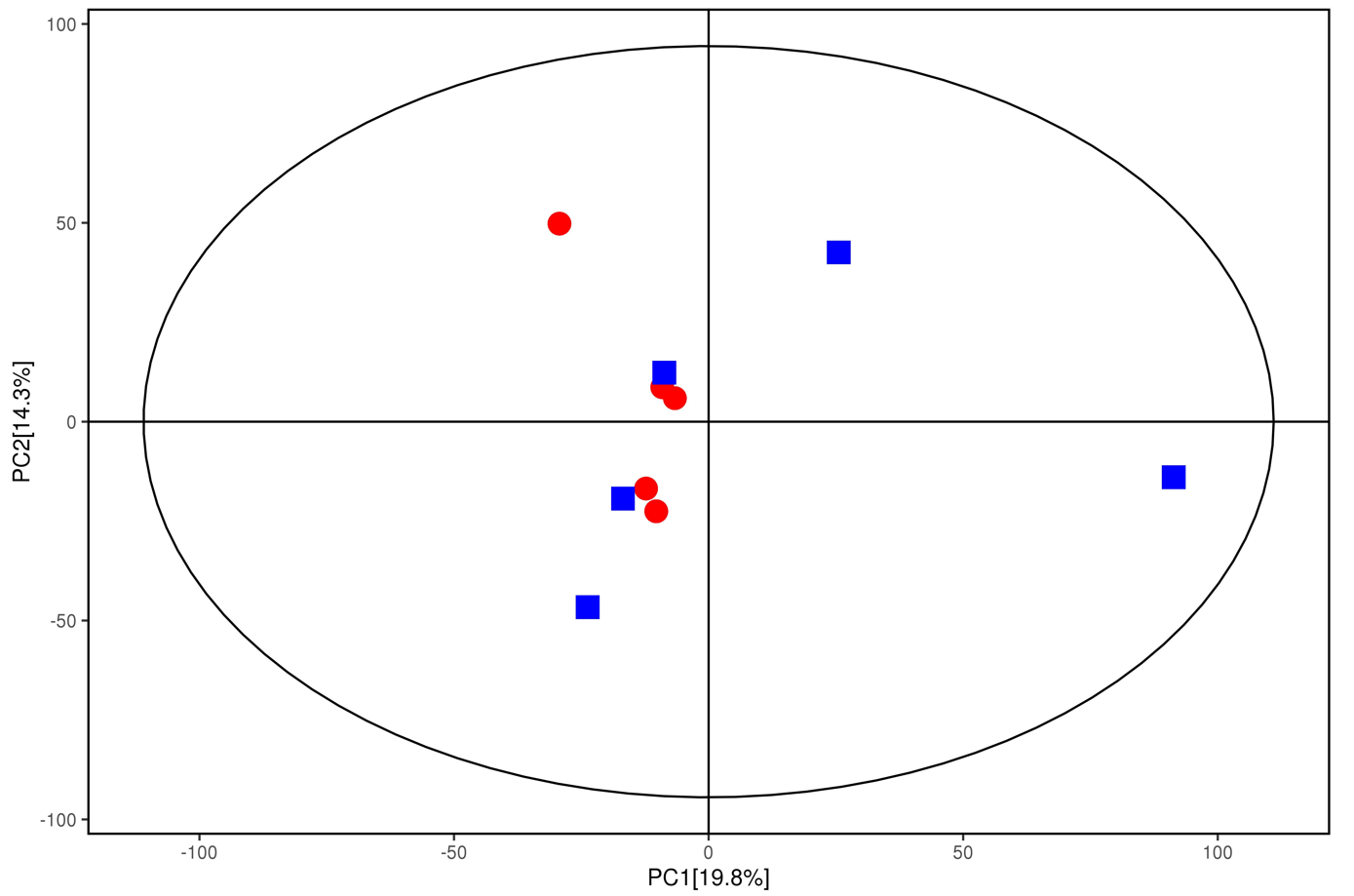


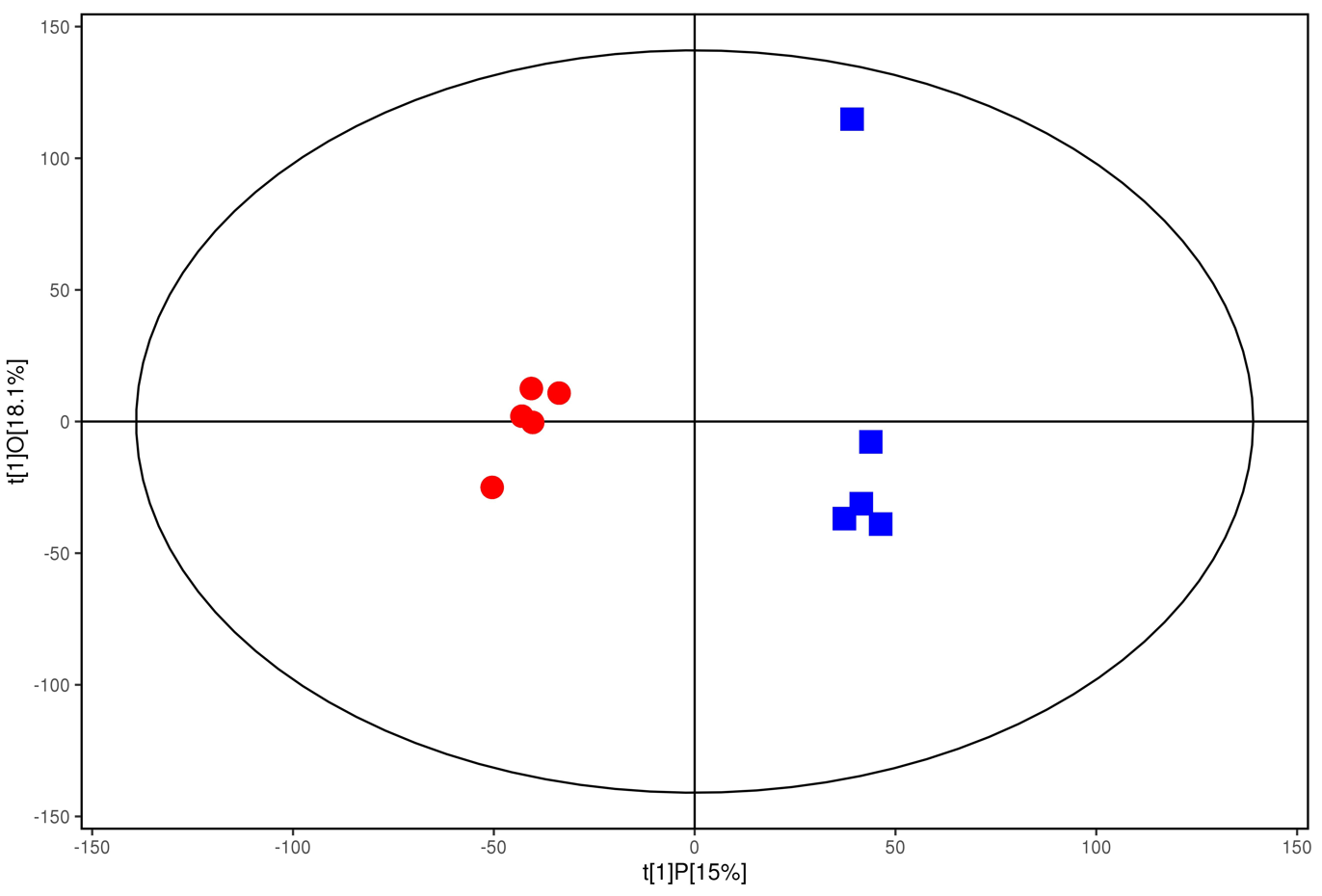


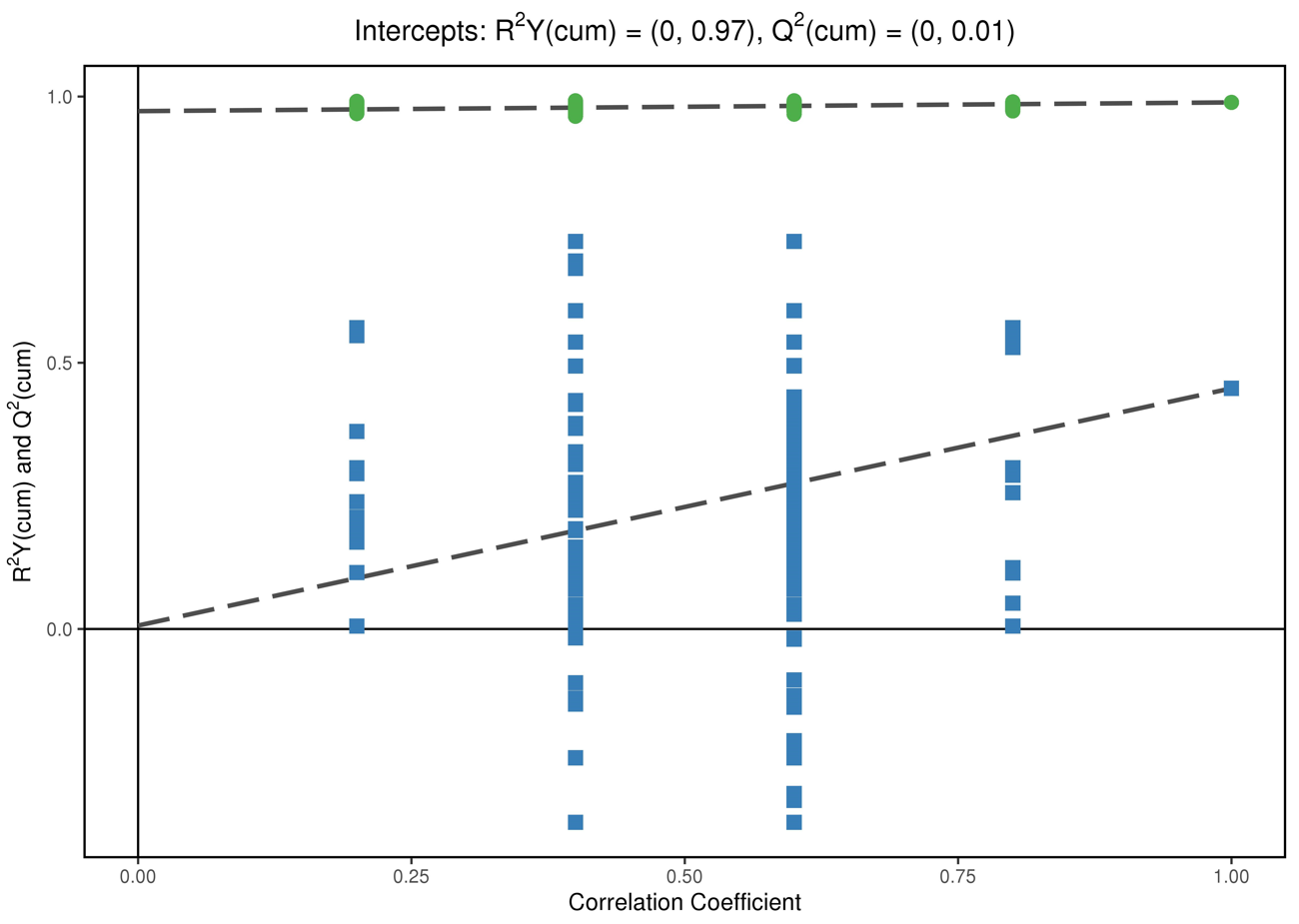


**Supplementary Figure S1.** Differential metabolites analysis in R and WT goosegrass individuals. a, Score plot (red dots for R and blue dots for WT) from PCA model. b, Score plot (red dots for R and blue dots for WT) from OPLS-DA model. c, Corresponding validation plot from OPLS-DA model. Correlation coefficient as the x-axis represents the replacement reservation degree of replacement test, and the y-axis represents the value of R^2^Y (green dots) and Q^2^ (blue square dots). The two dashes represent the regression lines of R^2^Y and Q^2^, respectively.
