# Supplementary material for: Vegetative and Fecundity Fitness Benefit Found in a Glyphosate-Resistant Eleusine indica Population Caused by 5-Enolpyruvylshikimate-3-Phosphate Synthase Overexpression
Source: Front Plant Sci. 2021 Nov 19;12:776990. doi: 10.3389/fpls.2021.776990 (PMC8639585; doi:10.3389/fpls.2021.776990)
Supplement: Supplementary file 1 [file Data_Sheet_1.zip › Supplementary Figure S2.DOCX]

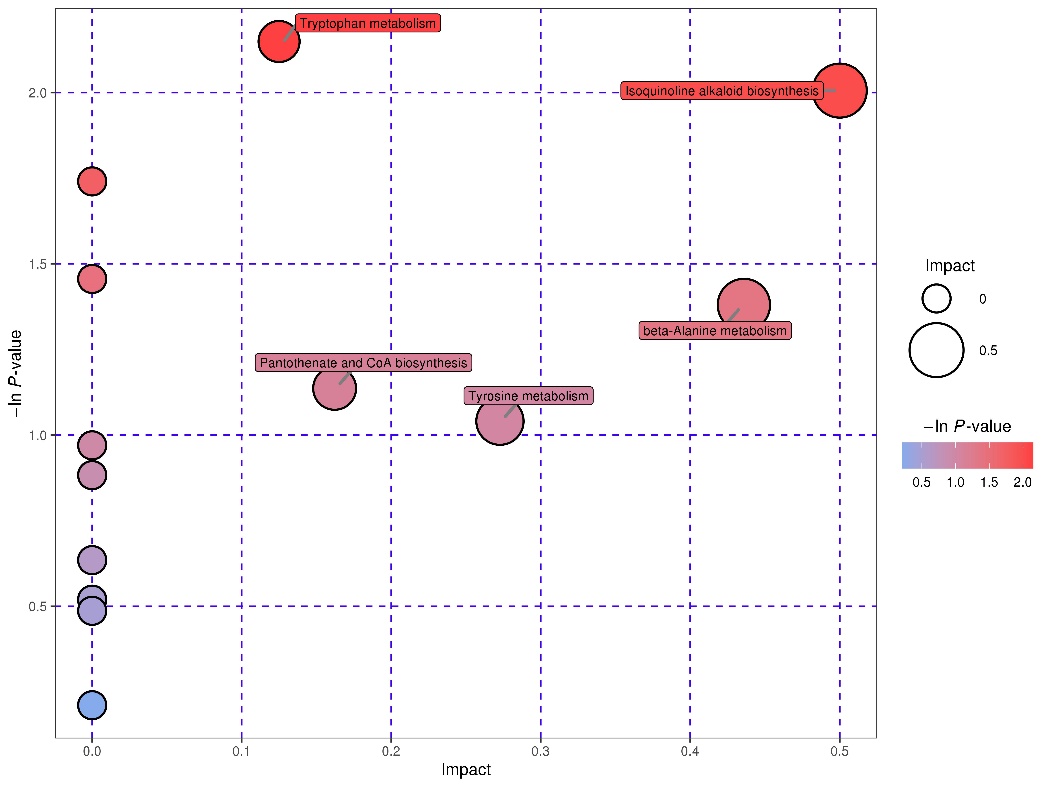


**Supplementary Figure S2.** Differential metabolites analysis in R and WT goosegrass individuals by pathway.
