# Supplementary material for: Vegetative and Fecundity Fitness Benefit Found in a Glyphosate-Resistant Eleusine indica Population Caused by 5-Enolpyruvylshikimate-3-Phosphate Synthase Overexpression
Source: Front Plant Sci. 2021 Nov 19;12:776990. doi: 10.3389/fpls.2021.776990 (PMC8639585; doi:10.3389/fpls.2021.776990)
Supplement: Supplementary file 1 [file Data_Sheet_1.zip › Supplementary Table S1.DOCX]

Supplementary Table S1. The differentially expressed metabolites between R and WT individuals.

| ID | MS2 name | MS2 score | VIP value | *P* value | Log_2_ fold change |
| --- | --- | --- | --- | --- | --- |
| 760 | Coriandrone B | 0.46 | 2.07 | 0.005 | 1.32 |
| 182 | 2',4',6'-Trihydroxyacetophenone | 0.62 | 2.23 | 0.000 | 1.14 |
| 172 | Anatabine | 0.96 | 2.08 | 0.014 | 1.06 |
| 90 | 6-Methylquinoline | 0.99 | 2.13 | 0.009 | 1.04 |
| 368 | Orientin | 0.80 | 2.18 | 0.004 | -1.47 |
| 90 | Cyanidin 3-glucoside | 0.95 | 2.05 | 0.002 | -1.46 |
| 260 | Diosmetin 7-neohesperidoside | 0.90 | 2.17 | 0.006 | -1.44 |
| 110 | Kaempferide | 0.90 | 1.93 | 0.004 | -1.43 |
| 249 | Luteolin 7-glucoside | 0.90 | 2.21 | 0.001 | -1.40 |
| 290 | (2R,3R)-2,3-Butanediol | 0.87 | 2.43 | 0.000 | -1.36 |
| 67 | L-Norleucine | 0.98 | 1.96 | 0.030 | -1.36 |
| 119 | 5,7-dihydroxy-2-(4-hydroxy-3,5-dimethoxyphenyl)-4H-chromen-4-one | 0.87 | 1.47 | 0.020 | -1.32 |
| 348 | 4-Hydroxycinnamoylagmatine | 0.82 | 1.30 | 0.010 | -1.30 |
| 192 | Licoagroside A | 0.95 | 1.70 | 0.048 | -1.29 |
| 174 | Diosmetin | 0.96 | 2.18 | 0.003 | -1.27 |
| 88 | Uric acid | 0.96 | 1.92 | 0.026 | -1.26 |
| 20 | Mulberrin | 1.00 | 1.96 | 0.006 | -1.25 |
| 271 | Quinone | 0.89 | 2.06 | 0.001 | -1.24 |
| 149 | 2-(4-Methyl-5-thiazolyl)ethyl octanoate | 0.97 | 2.39 | 0.001 | -1.22 |
| 89 | Bakers yeast extract | 0.99 | 2.28 | 0.003 | -1.21 |
| 372 | xi-8,9-Dehydrotheaspirone | 0.80 | 2.15 | 0.033 | -1.19 |
| 617 | Sumiki's acid | 0.61 | 1.61 | 0.013 | -1.18 |
| 620 | 1,4,5-Naphthalenetriol | 0.60 | 1.86 | 0.044 | -1.13 |
| 406 | 3-(5-Methyl-2-furanyl)butanal | 0.77 | 2.34 | 0.006 | -1.13 |
| 33 | Isopimaric acid | 1.00 | 1.33 | 0.046 | -1.10 |
| 256 | Racemethionine | 0.90 | 2.23 | 0.003 | -1.09 |
| 57 | Biochanin A | 0.98 | 1.93 | 0.001 | -1.09 |
| 122 | 4-Hydroxy-2-butenoic acid gamma-lactone | 0.98 | 2.28 | 0.001 | -1.08 |
| 154 | 3-Hydroxybutyric acid | 0.76 | 1.85 | 0.003 | -1.05 |
| 455 | 6''-Malonylcosmosiin | 0.74 | 1.17 | 0.010 | -1.02 |
